# Sanitary Affairs in South Africa

**Published:** 1901-08-31

**Authors:** 


### SANITARY AFFAIRS IN SOUTH AFRICA.

A COMMUNICATION from a regimental officer in South Africa appeared in the *Times* on the 24th inst., which shows how far those who are responsible for the management of affairs in South Africa are even yet from giving to matters of sanitation that full recognition which must be given to them if a stop is to be put to that constant draining of our forces by disease which is still going on. The losses caused by disease, and especially by enteric fever, soon after our occupation of Bloemfontein, were so excessive that by contrast the condition left when the great epidemic passed away seemed infinitely satisfactory. And it has been accepted as such. As a fact it is infinitely unsatisfactory. Although in the past summer there has been no epidemic in any way comparable with that which played such havoc in April and May of last year, still from December, 1900, to May, 1901, the death-rate and the invaliding roll were terribly heavy; in every week throughout that period "the number of officers and men lost or disabled by sickness was equal to the casualty list of a fairly large battle;" even now a considerable number of lives are being lost every week from enteric fever; and so far as it is possible to prophesy regarding any sublunary affairs it seems quite certain that unless more care be taken regarding certain matters which are quite elementary so far as common sanitary knowledge is concerned, we shall soon be just in the old hole again. Notwithstanding all that we know about the necessity of boiling all drinking water, and the periodical issue of "rather vague orders" on the subject, we are told that those on whom the responsibility is fixed have still "most inadequate means" of carrying such orders into effect. Not even yet, apparently, have arrangements been made at all railway stations to supply pure drinking water to troops passing through. Then as to the disposal of refuse we are told that anyone with average power of sight and smell who cares to loiter round any cantonment or camp can see what need there is for improvement. This, it is added, "is not a question of ignorance but of sheer neglect," and it is suggested—a great point coming from a regimental officer—that all stations and posts should be inspected at uncertain times by sanitary inspectors of good standing, and that these reports, "whether satisfactory or otherwise, should be taken into consideration by the superior authorities in their estimates of the military virtues of the officers responsible." This really hits at the root of the difficulty. Command is the appanage of the fighting branches of the army. Suggestion and advice alone may be given by the "doctors." The proposal that the extent to which those in command of a camp or station follow the expert advice of the "doctor" in regard to sanitary matters, should be taken into account in estimating an officer's military efficiency will no doubt be regarded as anathema by those in authority, and we are glad that it comes from a regimental officer. It is generally understood that officers have been, and are to be, dismissed from the service in cases where they have failed, or may fail, tactically or strategically as leaders of men, and this writer very properly asks "Are such also to be dismissed as prove indifferent or incapable in the matters of camp sanitation, or are special promotions or mentions to be made in the cases of commanding officers who, through careful attention to this tedious and uninteresting question, succeed in maintaining a high standard of health in their commands?" Let it once be understood that a commanding officer is at least as responsible for the health of his troops as a cavalry officer is for seeing to the condition of his horses, and that his promotion among other things depends upon the degree to which he and his medical adviser can, by mutual co-operation, keep the men in fighting condition, and we shall have gone a long way in solving one of the most serious problems in modern warfare. An army of sick men is a minus quantity.

